# Radiological Management of Chyle Leaks Following Oesophagectomy: A Systematic Review

**DOI:** 10.7759/cureus.96185

**Published:** 2025-11-06

**Authors:** Rahul Padmanabhan

**Affiliations:** 1 Hospital Medicine, Whittington Health NHS Trust, London, GBR

**Keywords:** chyle leak, chylothorax, lymphangiography, oesophagectomy, thoracic duct disruption, thoracic duct emobilisation

## Abstract

A chyle leak is a rare but potentially life-threatening complication following oesophagectomy. If untreated, it can result in hypovolaemia, malnutrition, electrolyte imbalance, and immunosuppression. Traditionally managed with surgical ligation, radiological interventions have emerged as less-invasive alternatives, though their comparative efficacy remains uncertain. This paper aims to systematically review the literature on the efficacy, safety, and clinical outcomes of radiological interventions for managing chyle leaks following oesophagectomy.

An electronic search of PubMed (Medline) was performed on 8th May 2024 using the terms “Chyle Leak,” “Chylothorax,” (accumulation of chyle within the pleural space), and “Oesophagectomy.” Eligible studies included patients with chyle leak post-oesophagectomy who underwent radiological interventions such as lymphangiography, thoracic duct embolisation (TDE), or thoracic duct disruption (TDD). Outcomes assessed included technical and clinical success rates, complications, and comparisons with surgical or conservative management. Both retrospective and prospective studies, case series, and case reports were included due to the limited data available related to the condition, specifically post-oesophagectomy. Data were synthesised qualitatively.

Of 448 studies identified, 38 met the inclusion criteria. The majority were retrospective reviews, case series, and case reports. Lymphangiography, with or without TDE/TDD, was the most frequently described intervention, demonstrating technical success rates exceeding 85% in most studies. Clinical resolution rates varied from 56% to 100%. Lymphangiography alone was often both diagnostic and therapeutic, with some studies reporting cure rates of up to 70%. Intranodal lymphangiography (INL) was often noted to have higher technical success than pedal lymphangiography (PL). Complications were uncommon and generally minor, though isolated cases of biliary fistula, haematoma, and Acute Respiratory Distress Syndrome (ARDS) were reported. Novel techniques such as MRI, CT-guided interventions, and lymphoscintigraphy demonstrated feasibility in case reports but lack high-powered validation.

Radiological interventions, particularly lymphangiography with or without TDE/TDD, are safe, minimally invasive, and increasingly effective alternatives to surgical ligation for managing chyle leaks after oesophagectomy. While the available evidence supports their early use, the literature is limited by heterogeneity, small sample sizes, and predominance of retrospective designs. Further prospective studies and standardised reporting are required to establish radiological intervention as the gold standard of management in chyle leak and to define its role relative to surgery.

## Introduction and background

Chyle leakage is a rare but potentially life-threatening complication following oesophageal resection, with a reported incidence in the literature between 1.1 and 9% [1-4]. 

Chyle is a lipid-rich, milky-white lymphatic fluid comprised of lymphocytes, vitamins, and chylomicrons (large lipoprotein particles consisting of triglycerides, cholesterol, and proteins, absorbed in the small intestine). This fluid is transported through the lymphatic system and returns to the venous circulation via the thoracic duct, the largest lymphatic vessel in the body, transporting between 1.5-4 L of chyle a day in healthy adults [5,6]. 

The thoracic duct arises from the cisterna chyli, a dilated lymph channel located in the retroperitoneum anterior to the L1 and L2 vertebral columns. The duct enters the thorax at the aortic hiatus between the aorta and azygous vein, running along the right side of the thoracic vertebrae, crossing the midline behind the oesophagus between T6-T4, entering the left posterior mediastinum, and finally terminating into the left venous angle (the point at which the left internal jugular vein and left subclavian meet). This course accounts for 60-65% of cases, with partial or complete duplication of the thoracic duct accounting for the most anatomical variation [1,2,5]. 

Given the proximity of the thoracic duct to the oesophagus and anatomical variation, oesophageal resection yields a notable risk of damage to the structure and subsequent leakage of chyle into the pleural or abdominal cavities. Leakage of chyle into the pleural space is known as chylothorax. Loss of chyle from the lymphatic circulation can be life-threatening, leading to hypovolaemia, electrolyte disturbance, severe malnutrition, and immunosuppression if untreated [7,8]. 

Post-operative chylothorax most commonly presents with persistent and excessive drainage of milky-white fluid from a chest thoracostomy following initiation of enteral nutrition, or in the absence of a chest drain, large pleural effusion with consequent respiratory compromise. Confirmatory diagnosis relies on clinical assessment, supported by pleural fluid analysis and, in some instances, lymphangiography, though this can in itself be therapeutic. If available, lipoprotein analysis confirming chylomicrons is the gold standard. If not available, fluid analysis measuring cholesterol and triglyceride levels is used. Fluid triglyceride levels greater than 110 mg/dl with a cholesterol level of less than 200 mg/dl are highly suggestive of chylothorax [5,6,9]. 

Thoracostomy output of more than 400 ml per day is an early indication of chylothorax. Indeed, output rate and response to parenteral nutrition are prognostic factors and guide first-line treatment [5]. Though a well-recognised complication, there had long been no international consensus on the criteria for diagnosis or gold-standard management of chyle leakage. Indeed, the absence of such a standardised system not only pertained to chyle leakage but to defining complications and quality measures following oesophageal resection as a whole. To address this, The Esophageal Complications Consensus Group was formed. The following classification for chyle leaks following oesophagectomy was determined [10]: (1) Type 1: Treated with an enteric dietary modification, such as a medium-chain triglyceride (MCT) diet;(2) Type 2: Treated with total parenteral nutrition (TPN); and (3) Type 3: Treated with interventional or surgical therapy, or based on severity, it was classified into: (1) Type A: Less than 1 litre output per day; and (2) Type B: Greater than 1 litre output per day. 

As highlighted above, treatment can broadly be divided into conservative versus active management. Conservative management, including parenteral nutrition, medium-chain triglyceride enteral feeding, or cessation of enteral feeding, chest drainage, and in some instances octreotide/somatostatin, is considered first-line treatment for low-output chylothorax. High-output chylothorax or chylothorax refractory to conservative management warrants active management. The gold standard for this has traditionally been re-thoracotomy with surgical exploration and ligation of the duct. However, such intervention has its own drawbacks. It is highly invasive, and given the patient demographic of likely malnourished and immunocompromised high-risk post-operative patients, such an intervention is associated with notable morbidity. In addition, the aforementioned anatomical variance means that the thoracic duct cannot always be identified intra-operatively [1,8,9,11]. 

As such, radiological interventions such as pedal lymphangiography (PL), thoracic duct embolisation (TDE), and thoracic duct disruption (TDD) have been gaining increasing attention as an alternative treatment, promising better identification of anatomical landmarks and less risk of morbidity associated with surgery, though consensus has not yet been reached as to its comparative efficacy and feasibility against surgical intervention. 

Thus, this systematic review of the literature aims to evaluate the efficacy of radiological management of chyle leaks following oesophagectomy, aiming to synthesise the evidence base and inform evidence-based practice. 

## Review

Methods 

Search Strategy and Eligibility Criteria 

An electronic search was performed through the PubMed (Medline) database using the terms “Chyle Leak,” “Chylothorax,” and “Oesophagectomy” on 8th May 2024. The search parameters were narrowed to include only English-written papers in order to minimise the risk of misinterpretation arising from translation inaccuracies and because of limited resources for professional translation. No limits were set for study type, cohort size, study setting, or time frame at the time of first screening. All references from relevant systematic reviews were screened for additional studies. 

Following screening of titles and abstracts, papers deemed relevant to the research question, namely those outlining the efficacy of radiological management, specifically post-oesophagectomy, were assessed in full against inclusion criteria according to the Population, Intervention, Comparison, and Outcome (PICOS) framework [12]. Eligible studies included patients with chyle leak or chylothorax following oesophagectomy, assessing radiological interventions (such as lymphangiography with or without thoracic duct embolisation or disruption) compared with surgical or conservative management, and reporting outcomes on technical or clinical success and complications across any study design. 

Due to the literature predominantly comprising studies of chylothorax from various aetiologies and a scarcity of data focusing on the radiological management of chyle leak exclusively post-oesophagectomy, this paper incorporates such studies provided they clearly delineate outcomes post-oesophagectomy.

As such, exclusion criteria were: studies not written in English; studies that did not clearly separate and report outcomes for chylothorax occurring post-oesophagectomy; studies that did not assess radiological management, such as lymphangiography or thoracic duct interventions; and studies that, during title and abstract screening, were deemed irrelevant to the efficacy of radiological management following oesophagectomy.

Despite the recognised limitation of case studies in analysis due to low statistical power, this paper highlights insights drawn from them, owing to the insufficient research in this field. Consequently, the presentation of results is in the form of a qualitative analysis, reflecting the variability in study design, interventions, clinical endpoints, and subsequent presentation of data. 

Results 

The electronic search generated a total of 448 results. Twenty-seven duplications were removed, yielding a total of 421 publications. 

**Figure 1:**
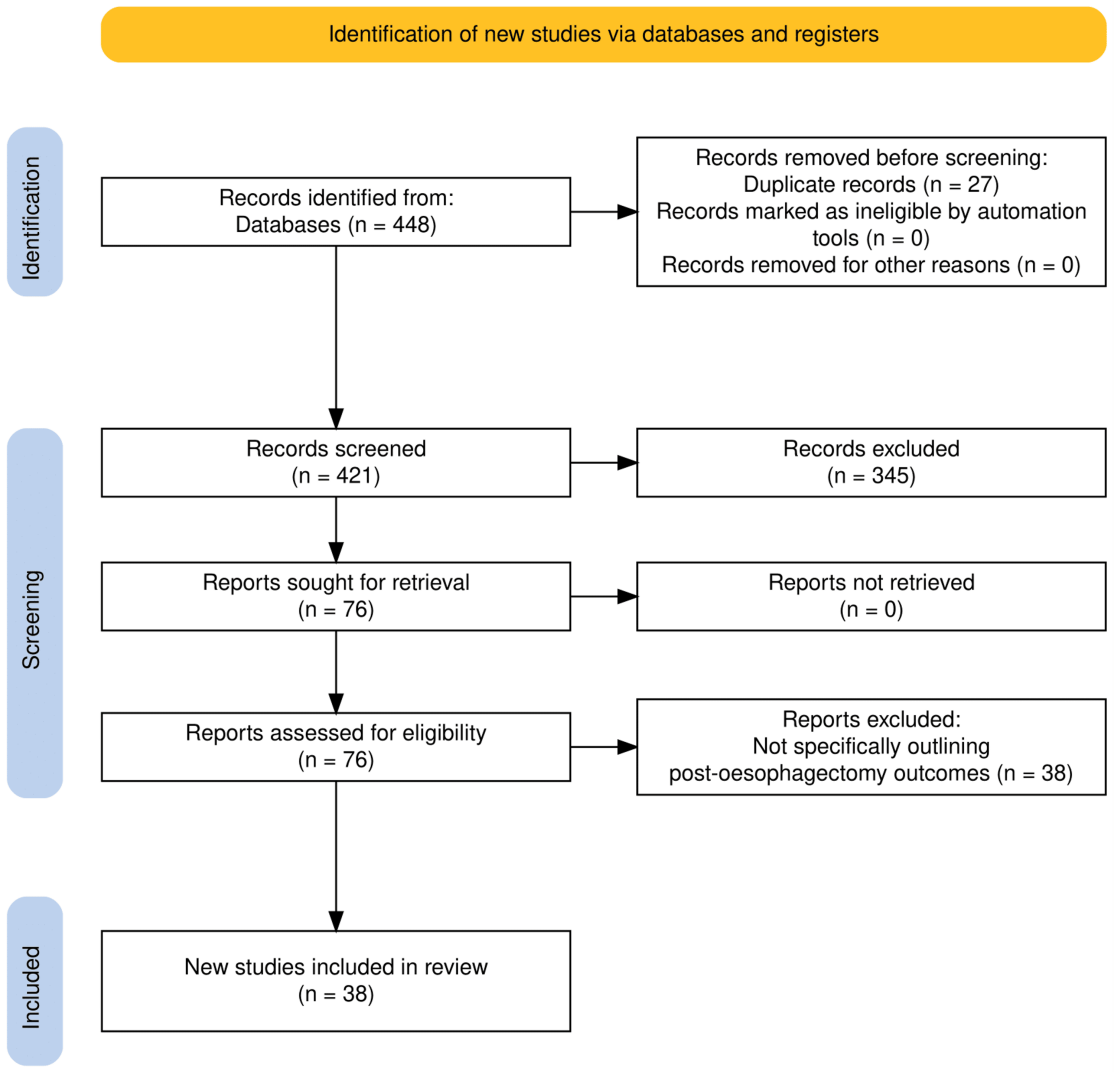
PRISMA flow diagram summarising the study selection process. A total of 448 records were identified, 27 duplicates removed, 421 screened, 76 full-text articles assessed, and 38 studies included in the qualitative synthesis. The PRISMA flow diagram tool was utilised [13].

The abstracts and titles of all 421 papers were screened for eligibility and relevance to the research question, after which 76 original studies relevant to radiological management of chyle leak following oesophagectomy remained. Seventy-six full-text manuscripts were assessed against inclusion criteria, and 38 were deemed eligible. The data for 38 original studies were extracted and synthesised as follows (Figure 1). 

Table 1 summarises the included papers, along with the radiological method, outcomes, and complications. 

**Table 1:** Summary of the published studies evaluating radiological interventions for chyle leak of mixed and post-oesophagectomy aetiologies PL: pedal lymphangiography; TDE: Thoracic Duct Embolisation; TDD: Thoracic Duct Disruption; INL: Intranodal Lymphangiography; MRI: Magnetic  Resonance Imaging; T2W: T2-weighted MRI; VATS: Video-assisted thoracoscopic surgery; ICG: Indocyanine Green; SPECT: Single-photon emission computed tomography; ARDS: Acute Respiratory Distress Syndrome.

Category / Study type	Author (Year)	Study design / N (Total study population)	Oesophagectomy Cases	Radiological technique(s)	Key findings / outcomes for post-oesophagectomy cases	Complications / notes
A. Mixed aetiologies (including oesophagectomy)	Cope et al. (1999) [14]	Prospective, 11	4 post-oesophagectomy	PL ± TDE	3/4 catheterised, of which: 1 cured due to catheterisation; 1 resolved conservatively, 1 with pleurodesis	None
	Cope (1998) [15]	Retrospective, 5	1 post-oesophagectomy	PL + TDE	1/1 cured	None
	Matsumoto et al. (2009) [8]	Retrospective, 9	4 post-oesophagectomy	PL	Leak identified in 3/4; all 4 cured after PL alone	None
	Pamarthi et al. (2014) [16]	Retrospective, 105	43 post-oesophagectomy	PL ± TDE/TDD	PL successful in 43/50; TDE/TDD successful in 28/50 (56%)	7 minor
	Bazancir et al. (2021) [17]	Retrospective, 17	11 post-oesophagectomy	INL + TDE	10/11 technically successful and cured	None
	Jeon et al. (2021) [18]	Retrospective, 48	18 post-oesophagectomy	INL + TDE	5/9 TDE success; ↓hospital stay (35.9→26.8d)	None
	Yannes et al. (2017) [19]	Retrospective, 57	14 post-oesophagectomy	INL ± TDE/TDD	12/14 cured (1 lymphangiography only, 7/8 TDE, 4/5 TDD)	1 minor haematoma
	Jardinet et al. (2021) [20]	Retrospective, 18	13 post-oesophagectomy	INL + Lipiodol	12/13 cured	None
B. Post-oesophagectomy only	Marthaller et al. (2015) [21]	Retrospective, 5	All post-oesophagectomy	INL/PL + TDE or CT-guided	4/5 cured; recommend early intervention	None
	Farran et al. (2021) [22]	Retrospective, 13	All post-oesophagectomy	Lymphangiography + TDE	Leak identified in all 10 attempted; 9/10 cured	1 biliary fistula
	Abe et al. (2016) [23]	Retrospective, 9	All post-oesophagectomy	PL or INL + Lipiodol	Leak identified in 8/9, 5/5 cured following lymphangiography only, 2 cured following lymphangiography + surgical management, 2 lymphangiography + pleurodesis.	None
	Liu et al. (2016) [24]	Case series, 3	All post-oesophagectomy	INL + Lipiodol	1 cured; 2 required ligation	None
	Lambertz et al. (2019) [4]	Case series, 4	All post-oesophagectomy	INL + TDE	3 cured with TDE, 1 with TDD	None
	Kariya et al. (2014) [25]	Case series, 3	All post-oesophagectomy	INL ± TDE	1 INL-only cure; 2 TDE cures	None
	Suetsugu et al. (2020) [5]	Case series, 3	All post-oesophagectomy	INL ± Ligation	1 INL-only cure; 2 ligation cures	None
	Ishida et al. (2019) [1]	Case report	1	Lymphangiography + pleurodesis	Leak cured	None; aberrant thoracic duct
	Atie et al. (2016) [9]	Case report	1	Lymphangiography + TDE	Leak cured	None
	Yamamoto et al. (2015) [11]	Case report	1	INL alone	Leak cured	None
	Schild & Hirner (2001) [26]	Case report	1	Translymphatic TDE	Leak cured	None
	Haneda et al. (2020) [27]	Case report	1	Lymphangiography + ligation	Leak cured	None; duplicated duct
	Sato et al. (2021) [28]	Case report	1	INL + retrograde TDE	Leak cured	None
	Shimakawa et al. (2017) [29]	Case report	1	Lymphangiography + ligation	Leak cured	Rare double thoracic duct
	Williams et al. (2021) [30]	Case report	1	TDE via chest tube	Leak cured	None
	Chang et al. (2017) [31]	Case report	1	MRI (T2W) + VATS ligation	Leak cured	None
	Tsuda et al. (2015) [32]	Case report	1	Lymphoscintigraphy	Leak cured	None
	Matsutani et al. (2014) [33]	Case report	1	ICG fluorescence + ligation	Leak cured	None
	Kaburagi et al. (2013) [34]	Case report	1	ICG fluorescence + ligation	Leak cured	None
	Das et al. (2015) [35]	Case report	1	Lymphoscintigraphy + SPECT/CT	Leak cured	None
	Kamiya et al. (2009) [36]	Case report	1	ICG fluorescence intraoperative	Leak cured	None
	Bybel et al. (2001) [37]	Case report	1	Lymphoscintigraphy	Leak cured conservatively	None
	Motoyama et al. (2005) [38]	Case report	1	MRI + ligation	Leak cured	None
	Kotani et al. (2012) [39]	Case report	1	Lymphoscintigraphy + SPECT/CT	Leak cured	None
	Barnidge et al. (2008) [40]	Case report	1	CT-guided drainage	Leak cured	None
	Onikubo et al. (2022) [41]	Case report	1	CT-guided INL (Lipiodol)	Leak cured	None
	Wang & Jiang (2022) [42]	Case report	1	Imaging-guided management	Leak resolved	None
	Taki et al. (2019) [43]	Case report	1	Lipiodol lymphangiography	ARDS from Lipiodol exposure	ARDS, rare complication
	Drabkin et al. (2020) [44]	Case report	1	Combined antegrade + retrograde TDE	Leak cured	None
	Farahnak et al. (2023) [45]	Case report	1	CT-guided microwave ablation	Leak cured	None
	Praveen et al. (2012) [2]	Case report	1	MRI + fluoroscopy guided TDE	Leak cured	None; MRI improved cisterna chyli visibility

Papers Assessing Radiological Management of Chyle Leak of Various Aetiologies but Delineating Outcomes for Oesophagectomy 

Seven retrospective studies and one prospective study assessed the radiological management of chyle leaks of a variety of aetiologies but importantly delineated post-oesophagectomy outcomes allowing for deduction of findings in this review. 

Cope, in 1998, conducted a retrospective review of five cases assessing the efficacy of PL and percutaneous embolisation in treating post-operative chylous effusions. One case of chyle leak post-oesophagectomy was included in the review and underwent successful PL, thoracic duct catheterisation, and subsequent percutaneous embolisation with cure, no complications were noted. This study represents the earliest evidence in the literature of the efficacy of utilising lymphangiography and embolisation for the treatment of post-operative chyle leak [15]. 

Indeed, Cope et al. conducted a prospective trial the following year, assessing the efficacy of PL and percutaneous embolisation in 11 cases of chylothorax, four of which occurred post-oesophagectomy. The thoracic duct was successfully catheterised following PL in three of these four patients. Of the successfully catheterised cases, a chyle leak was identified in one case, successfully embolised and cured. In another case no leak was identified, no management was performed, and the leak resolved with conservative management; in the third case, though no leak was identified through lymphangiography, the thoracic duct was embolised successfully, however, this had no noticeable effect, and the patient required pleurodesis for resolution of the leak. A chyle leak was successfully opacified in the case of unsuccessful TD catheterisation, however, the patient suffered from sepsis and died from cardiac arrest three weeks later. No procedural complications were noted, with the authors concluding that wider utilisation of thoracic duct catheterisation and embolisation is warranted for uncontrolled postsurgical chylothorax, especially when patients are too unwell to undergo a second operation. Though they acknowledged the limit of drawing conclusions from a small cohort, they noted the technique to be a potentially safe and effective alternative treatment for persistent chylothorax which should be attempted before considering surgical ligation [14]. 

Studies since then appear to echo this sentiment. Pamarthi et al. performed a retrospective review assessing the efficacy of TDE or TDD in addition to PL in treating 105 patients suffering from an iatrogenic chyle leak. TDD was performed when embolisation was not technically possible. Forty-three patients within this cohort suffered a chyle leak following oesophagectomy, and 50 radiological procedures were performed within this group. PL was technically successful in 43/50 procedures, TDE was successful in 27/43 patients, with TDD performed successfully in one more, bringing the total success rate to 28/43 patients or 28/50 radiological procedures performed in the post-oesophagectomy cohort. Seven minor complications were noted in this group [16]. 

Matsumoto et al. performed a retrospective review assessing the efficacy of PL in both identifying and treating post-operative chyle leak in nine cases, four of which followed oesophagectomy. Lymphangiography was successfully performed in all four cases, identifying the leak in three and successfully curing all four. No complications were noted. This study highlighted the utility of lymphangiography in not only identifying the chyle leak but as a curative intervention as well. Such results have been reproduced in the literature, suggesting that lymphangiography should be used as a first-line management in unremitting chylothorax prior to determining next steps [8]. 

The comparative efficacy of PL versus intranodal lymphangiography (INL) appears to be a well-discussed, though not formally assessed, comparison within the literature, with the suggestion that INL is a superior route for performing lymphangiography due to the procedure being technically more straightforward and consequently more effective. 

Bazancir et al. conducted a retrospective review of INL and TDE in treating 17 cases of chyle leak, 11 following oesophagectomy. In these 11 cases, lymphangiography was successful in 10 instances, with TDE successfully curing the leak in all 10 of these patients [17]. Jeon et al. similarly conducted a retrospective review of INL and TDE in 48 cases of chyle leak, 18 post-oesophagectomy. In nine cases attempted within the post-oesophagectomy cohort, TDE was successful in five instances. Interestingly, this study compared two time periods: Period one of management of chyle leak prior to the availability of TDE at that centre, and period two, after introduction of TDE. In period one, for the post-oesophagectomy cohort, four patients were treated with conservative measures of low-fat diet or cessation of feeding, and four were treated with surgical ligation. Mean hospital stay was 35.9 days ± 25.9, and mean time to oral feeding was 17.3 days ± 9.1. In period two, within the post-oesophagectomy cohort, 11 patients were treated with conservative measures of low-fat diet or cessation of feeding, two patients underwent surgical ligation, and five were successfully treated with TDE. Mean hospital stay was 26.8 days ± 17.3, and mean time to oral feeding was 12.8 days ± 6.1. They noted that due to the burden of surgical ligation, conservative treatment continued for more than a week in cases of chyle leak refractory to surgical intervention. Conversely, TDE was utilised as an alternative to surgical ligation for refractory chyle leak in period two. When successful, this allowed for earlier intervention and thus resolution of chyle leak, leading to shorter hospital stay and time to oral feeding. Though not within the scope of this review, the study also found that TDE had a higher success rate in curing chyle leak following lung cancer surgery as opposed to oesophagectomy due to the latter being more technically challenging [18]. Both studies add to the evidence base for the utility of INL and TDE in treating chyle leak following oesophagectomy. 

Yannes et al. conducted a retrospective review assessing the efficacy of INL with or without TDE or TDD in 57 cases of chyle leak, 14 of which were post-oesophagectomy. Lymphangiography was successfully performed in all post-oesophagectomy cases. One case resolved with lymphangiography only; TDE was successful in curing seven of eight cases attempted, and TDD was successful in four out of five. One case had a minor complication of a haematoma between the aorta and vertebral body. This review reinforces the efficacy of INL in conjunction with TDE or TDD, but also highlights the potential efficacy of lymphangiography as a curative intervention itself, further suggesting that lymphangiography should be utilised as a first step in management upon which further interventions can be employed if required [19]. 

Lymphangiography as a sole intervention in treating chyle leak was assessed by Jardinet et al., who retrospectively reviewed the efficacy of INL with high-dose ethiodised oil in 18 cases of chyle leak, 13 of which were post-oesophagectomy. A cure was successfully achieved in 12 of these post-oesophagectomy cases, concluding this to be a safe and effective procedure for management of refractory post-surgical chylothorax [20]. 

Studies Assessing Efficacy of Radiological Interventions Solely Post-Oesophagectomy 

Unfortunately, there is a paucity of high-powered studies assessing the efficacy of radiological interventions in the management of chyle leak solely post-oesophagectomy, with the majority of literature comprised of case series and studies. Though not controlled trials, these studies cover a wide variety of radiological interventions with promise in treating chyle leak effectively and can provide the basis upon which further trials are based. Consequently, these studies have been included in this review, and their findings are summarised in Table 1. 

Three retrospective studies, four case series, and 23 case studies have been included in the evaluation. Marthaller et al. conducted a retrospective review of five patients with chyle leak following oesophagectomy. Two patients were treated with INL and percutaneous TDE, two with PL and percutaneous TDE, and one with CT-guided direct cannulation. All procedures were technically successful and identified the leak. Four of five cases successfully treated the leak, with one case of PL unsuccessful. They recommend considering percutaneous intervention as early as one week after confirmation of persistent chyle leak to allow sufficient time for closure and to minimise metabolic and immunologic consequences that result from leak. They highlighted INL as a novel cannulation technique which can replace traditional PL and concluded that this technique can treat most TD disruptions regardless of cause. They advise that it should be considered first-line therapy in post-oesophagectomy chyle leak and is likely to replace traditional thoracotomy with ligation in the future [21]. 

Farran et al. conducted a retrospective review of 274 cancer-related oesophagectomies, of which 13 had a chyle leak. Ten cases were treated with lymphangiography and TDE. Lymphangiography was successfully performed in all ten cases, successfully identifying the leak, and a cure was achieved in nine cases. One case presented with a biliary fistula following the procedure [22]. 

Abe et al. performed a retrospective review of the efficacy of lymphangiography with lipiodol in treating nine cases of chyle leak following oesophageal resection. PL was successfully performed in six of the six cases, and IL in two of three cases. In total, the leak was identified in eight of nine instances. Following lymphangiography, daily outputs of less than 500 ml were observed in five cases, all of which subsequently healed conservatively, and daily outputs of more than 500 ml were observed in the remaining four. Of these four, one patient had leakage from the main trunk of the thoracic duct and underwent ligation, one patient had anatomical variants and underwent surgical treatment, and the remaining two had no anatomical variants and were successfully treated with pleurodesis. The overall success rate of treatment in this review was 100%, further adding to the evidence base of the benefit of lymphangiography as a treatment modality in itself, but also as a first step in management of identifying the location of chyle leak with a view for further intervention with different methods if required [23]. 

All four case series assessed the efficacy of INL either on its own or in conjunction with another treatment modality in treating chyle leak. Liu et al. described three cases of chyle leak treated with INL using lipiodol. Lymphangiography was performed successfully and identified the leak in all three cases. The leak was cured in one out of three cases using this method, and two cases were cured with surgical ligation following INL, and no complications were noted. The authors concluded that INL with lipiodol was an “easily performed procedure with high diagnostic and therapeutic value for postoperative chylothorax,” mirroring the wider conclusions drawn in the literature [24]. 

Lambertz et al. assessed the efficacy of INL with direct embolisation using lipiodol in four cases. INL was successfully performed and identified the leak in all four cases. Cure was achieved in three of the cases using embolisation, with the remaining case treated through disruption. No complications were reported. They concluded that the procedure was “feasible and safe,” indicated for high-output chyle leaks, and advised that it be performed early after diagnosis of the complication [4]. 

Kariya et al. described three instances of INL with or without TDE used to treat chyle leak. Lymphangiography was successfully performed in all and identified the leak. Lymphangiography as a sole intervention cured one of the three cases, with TDE curing the remaining two [25]. Suetsugu et al. described three cases of INL with lipiodol ± ligation used to treat chyle leak. Similarly, lymphangiography was successfully performed and identified the leak in all three cases, curing the leak as a sole intervention in one case. Ligation successfully cured the remaining two cases [5]. 

The data for case studies included within this review can be seen in greater detail in Table 1. Although it is noted that the risk of publication bias is greater in case studies than retrospective reviews or controlled trials, the numerous case studies provide evidence for the success centres have had in utilising radiological methods for the management of chyle leak [1,9,11,26-45]. 

The case studies included a variety of cases outlining the success of PL or INL with or without additional treatment modalities such as embolisation, ligation, or focal pleurodesis. Of note, there are a few case studies which describe variations in aforementioned radiological methods through use of CT or MRI and, in some instances, lymphoscintigraphy with radiolabelled markers. 

Chang et al. and Motoyama et al. both highlighted the efficacy of MRI in identifying the site of chyle leak following oesophagectomy. The former case successfully utilised video-assisted thoracoscopic thoracic duct ligation to treat the chyle leak [31,38]. Praveen et al. described a case study of combined heavily T2-weighted (T2W) MRI and fluoroscopy as guidance for thoracic duct puncture for a case of chyle leak following oesophagogastrectomy. They noted that heavily TW2 MRI has a higher sensitivity in identifying cisterna chyli than is reported for lymphangiography and CT. In addition, they note its major advantages to include avoidance of invasive lymphangiography, better visualisation of TD and lymphatic channels than bipedal lymphangiography, 3D visualisation, and its speed [2]. These studies suggest that further research into the use of MRI as a radiological method for identification and management of chyle leak is warranted. 

Barnidge et al. and Onikubo et al. both outlined the successful use of CT guidance, the former for percutaneous drainage and the latter for lymphangiography through the para-aortic lymph nodes [40,41]. 

Farahnak et al. described the immediate success of microwave ablation through CT fluoroscopic guidance in treating refractory chylothorax following two failed attempts of interventional radiology (IR) embolisation. The authors acknowledge a possibility that the chyle leak could have resolved over time without intervention and thus further research is required to assess the utility of this approach [45]. 

Matsutani et al. and Kaburagi et al. both discussed the successful usage of indocyanine green (ICG) with lymphangiography as a fluorescent navigation method to identify the site of chyle leak for ligation [33,34]. Kamiya et al. described its use intraoperatively for detection of chylothorax fistula site [36]. 

Four cases described the use of lymphoscintigraphy through a variety of radiolabelled markers and in conjunction with varying modalities and treatments such as ligation, pleurodesis, or CT imaging [32,35,37,39]. The reported success of the treatment through all methods reflected, on a wider level, the extensive range of interventions used in the literature and the large range of modifiable treatment options currently utilised in treating a chyle leak. 

Discussion 

A chyle leak is a rare but life-threatening complication following oesophagectomy. Traditionally, the gold standard treatment for a post-operative chyle leak has been thoracotomy with surgical ligation; however, such intervention carries a significant risk due to its invasive and burdensome nature for patients who are already deconditioned following oesophagectomy and loss of chyle. 

In recent years, radiological interventions have gained prominence as first-line strategies owing to their capacity to both characterise and potentially treat chyle leak with lower physiological burden. Within this paradigm, lymphangiography has emerged as a pivotal first-line intervention. Across heterogeneous studies, it demonstrated high technical success with reliable localisation of the leak and a favourable safety profile. Of note, its minimally-invasive nature permitted earlier intervention than surgical exploration, thereby facilitating timely, targeted treatment and, in some instances, directly promoted leak cessation. Lymphangiography also functions as an enabling step for additional intervention, most commonly TDE or TDD, and less frequently adjunctive procedures such as pleurodesis or ligation when required. 

Early consensus in the literature suggests INL to be operationally superior to PL, with a higher technical success rate. However, few studies directly compare the two interventions in a controlled setting. Further research could be considered in this area to strengthen the evidence base. 

As a definitive therapy, TDE/TDD has become the most employed alternative to surgical ligation. Reported clinical resolution rates overlap with those described for ligation, but the overall risk profile of TDE/TDD is generally more acceptable, with mostly minor complications and rare serious adverse events. By contrast, technical success at ligation does not always translate into clinical resolution, and peri-intervention morbidity and mortality seem to be higher, particularly when surgery is undertaken early in deconditioned patients. These trends support an imaging-led, stepwise approach: conservative management followed by early INL-guided lymphangiography (diagnostic with potential therapeutic effect), escalation to TDE/TDD where indicated, and reservation of open ligation for refractory cases, unfavourable anatomy, or where interventional options are unavailable. 

Despite the literature suggesting this, the evidence base remains limited due to the condition’s rare and heterogeneous nature with substantial variation in anatomy, output, response to conservative management, timing of intervention, contrast/embolic agents, and endpoints. The literature is dominated by retrospective series and case reports, with few direct comparisons between radiological strategies and surgical ligation and no randomised trials. Selection and publication bias are therefore of concern, and meta-analysis is not feasible at this stage. Novel imaging approaches, including CT- or MR-based techniques and lymphoscintigraphy, show promise in case-level data but require prospective evaluation before broader adoption. 

Accordingly, only cautious conclusions can be drawn. The available data support early lymphangiography with selective use of TDE/TDD. This is a pragmatic default that balances efficacy with patient safety, while recognising that surgical ligation retains a role in non-responders or when anatomical considerations prevent radiological treatment. To strengthen the evidence base and standardise practice, future work should prioritise a prospective registry with uniform reporting parameters, and where feasible, prospective comparative studies to clarify timing, patient selection, and optimal sequencing of radiological versus surgical interventions. 

## Conclusions

Radiological management of chyle leak following oesophagectomy appears to be a safe and minimally invasive alternative to surgical ligation. Techniques such as lymphangiography with or without TDE or TDD show encouraging technical and clinical success, potentially enabling earlier and less invasive intervention. However, current evidence is constrained by small, retrospective studies and heterogeneous reporting. Further prospective, multi-centre research and standardised outcome measures are required to better define efficacy and establish best practice in this evolving field.
